# The lncRNA CASC2 Modulates Hepatocellular Carcinoma Cell Sensitivity and Resistance to TRAIL Through Apoptotic and Non-Apoptotic Signaling

**DOI:** 10.3389/fonc.2021.726622

**Published:** 2022-01-25

**Authors:** Jichun Sun, Hongbo Xu, Zhao Lei, Zhiqiang Li, Hongwei Zhu, Zhen Deng, Xiao Yu, Xiaoxin Jin, Zhi Yang

**Affiliations:** ^1^ Department of Hepatobiliary and Pancreatic Surgery, The Third Xiangya Hospital, Central South University, Changsha, China; ^2^ Department of Vascular Surgery, The Third Xiangya Hospital, Central South University, Changsha, China; ^3^ Department of General Surgery, The Second Xiangya Hospital, Central South University, Changsha, China; ^4^ Department of Colorectal & Anal Surgery, General Surgery, Xiangya Hospital, Central South University, Changsha, China; ^5^ National Clinical Research Center for Geriatric Disorders, Xiangya Hospital, Central South University, Changsha, China

**Keywords:** hepatocellular carcinoma, TNF-related apoptosis-inducing ligand (TRAIL), lncRNA CASC2, miR-18a, receptor-interacting serine/threonine-protein kinase 1 (RIPK1), the NF-κB signaling

## Abstract

The immune cytokine tumor necrosis factor-related apoptosis-inducing ligand (TRAIL) has been widely concerned as a tumor therapy because of its ability of selective triggering cancer cell apoptosis; nevertheless, hepatocellular carcinoma (HCC) exhibits acquired resistance to TRAIL-induced apoptosis. In the present study, tumor-suppressive lncRNA cancer susceptibility candidate 2 (CASC2) was downregulated in HCC tissues and cell lines; HCC patients with lower CASC2 expression predicted a shorter overall survival rate. *In vitro*, CASC2 overexpression dramatically repressed HCC cell proliferation and inhibited cell apoptosis; *in vivo*, CASC2 overexpression inhibited subcutaneous xenotransplant tumor growth. CASC2 affected the caspase cascades and NF-κB signaling in TRAIL-sensitive [Huh-7 (S) and HCCLM3 (S)] or TRAIL-resistant cell lines [Huh-7 (R) and HCCLM3 (R)] in different ways. In Huh-7 (S) and HCCLM3 (S) cells, CASC2 affected cell apoptosis through the miR-24/caspase-8 and miR-221/caspase-3 axes and the caspase cascades. miR-18a directly targeted CASC2 and RIPK1. In Huh-7 (R) and HCCLM3 (R) cells, CASC2 affected cell proliferation through the miR-18a/RIPK1 axis and the NF-κB signaling. RELA bound to CASC2 promoter region and inhibited CASC2 transcription. In conclusion, CASC2 affects cell growth mainly *via* the miR-24/caspase-8 and miR-221/caspase-3 axes in TRAIL-sensitive HCC cells; while in TRAIL-resistant HCC cells, CASC2 affects cell growth mainly *via* miR-18a/RIPK1 axis and the NF-κB signaling. These outcomes foreboded that CASC2 could be a novel therapeutic target for further study of HCC-related diseases.

## Introduction

Hepatocellular carcinoma (HCC) is one of the most common malignant tumors worldwide, and its high fatality rate and high incidence are a serious threat to public health (1). Tumor necrosis factor-related apoptosis-inducing ligand (TRAIL) is a member of the tumor necrosis factor (TNF) family and widely expresses in a variety of tissues; due to the difference in sensitivity to TRAIL between normal cells and tumor cells, TRAIL can selectively induce apoptosis of tumor cells but almost has no killing effect on normal cells; hence, TRAIL is considered a promising antitumor agent (2–4). However, in phase II clinical trials, the efficacy of TRAIL preparations has not yet reached expectations, and many types of tumor cells, including liver cancer, are resistant to TRAIL (5, 6). Therefore, exploring the mechanism underlying HCC resistance to TRAIL would provide novel directions in the clinical applications of TRAIL therapy.

According to previous studies, TRAIL acts on sensitive and resistant tumor cells through different signaling pathways (7). Upon TRAIL binding with death receptor 4/5 (DR4/5), the homotrimer recruits FADD (fas-associated death domain) and procaspase-8/10 to form the DISC (death-inducing signaling complex) (8–10); thus, caspase-8/10 cleavage in the DISC leads to the activation of downstream caspase-3/6/7, inducing apoptosis (1, 11). On the other hand, activated caspase-8 promotes the expression of RIPK1 (receptor-interacting serine/threonine-protein kinase 1), inhibits the activation of NF-κB pathway, and inhibits the proliferation of tumor cells (12). However, since death receptors showed to be expressed ubiquitously in both cancer and non-cancerous cells (13, 14), HCC exhibits acquired resistance to TRAIL-induced apoptosis (15–18). In TRAIL-resistant cell lines (9–11), TRAIL recruits TNF receptor 1 associated death domain protein (TRADD)/TNF receptor-associated factors (TRAFs) through the receptor DR4/DR5 receptor to activate the NF-κB pathway and promote cell proliferation, while activated NF-κB targeted and inhibited the caspase pathway, thereby inhibiting tumor cell apoptosis. Therefore, inhibiting the activation of the NF-κB pathway to amplify the caspase cascades might be the key to reversing TRAIL resistance of HCC.

For the past few years, non-coding RNAs, including long non-coding RNAs (lncRNAs) and miRNAs, were found to act as significant regulatory factors in the development of cancers and other diseases, and it has been reported that lncRNAs and miRNAs are implicated in the chemotherapy resistance to multiple agents (19, 20). Notably, many studies have evaluated the efficacy of TRAIL in combination with miRNAs and lncRNAs for HCC treatment (16, 21–23). In our previous study, we found that miR-24 and miR-221 could suppress the expression level of caspase-8/3, leading to HCC TRAIL resistance; nevertheless, cancer susceptibility candidate 2 (CASC2), a widely known antitumor lncRNA (24, 25), could inhibit the effects of miR-24 and miR-221 *via* acting as their “sponge,” suggesting that CASC2 could enhance the sensitivity of HCC cells to TRAIL treatment through CASC2/miR-24/miR-221 axis (26). LncRNA CASC2, located at chromosome 10q26, was first identified to be dysregulated in endometrial carcinoma (27). CASC2, as a tumor suppressor, was subsequently discovered to play a crucial part in multiple tumor diseases, including pancreatic cancer (28), papillary thyroid cancer (29), cholangiocarcinoma (30), glioma (31), and so on. Given the critical role of the NF-κB pathway in HCC resistance to TRAIL, identifying more lncRNA CASC2/miRNA axes modulating the NF-κB pathway in HCC cells might provide novel targets for restoring TRAIL sensitivity.

Herein, the expression of lncRNA CASC2 was determined in HCC tissue samples and cell lines. Then, the *in vitro* effects of CASC2 on HCC cell proliferation and apoptosis and the *in vivo* effects of CASC2 on subcutaneous xenotransplant tumor growth were examined. The key factors of the caspase cascades (RIPK1, caspase-8, and caspase-3) and NF-κB signaling (IKKβ, p-IκBα, and p-p65) were monitored in TRAIL-sensitive cell lines [Huh-7 (S) and HCCLM3 (S)] and TRAIL-resistant cell lines [Huh-7 (R) and HCCLM3 (R)], and the effects of CASC2 on two signaling pathways were investigated in TRAIL-sensitive and TRAIL-resistant cell lines, respectively. Next, the effects of the CASC2/miR-24/caspase-8 and CASC2/miR-221/caspase-3 axes on TRAIL-sensitive cell [Huh-7 (S) and HCCLM3 (S)] apoptosis were investigated. Given the key role of RIPK1 in TRAIL therapy, miRNAs that might target CASC2 and RIPK1 were analyzed, and miR-18a was selected. The predicted bindings between CASC2 and miR-18a or between miR-18a and RIPK1 were validated. The dynamic effects of the CASC2/miR-18a/RIPK1 axis on the NF-κB signaling and TRAIL-resistant cell [Huh-7 (R) and HCCLM3 (R)] proliferation were investigated. Given that online tools predicted possible RELA binding sites on CASC2 promoter region, the predicted bindings were validated using dual-luciferase reporter and ChIP assays. Finally, the expression and correlation of key factors in tissue samples were investigated.

## Materials and Methods

### Clinical Sampling

Under the approval by the Research Ethics Committee of the Third Xiangya Hospital, samples were obtained from patients who had never received any therapies before sampling and signed written informed content in advance. A total of 15 HCC tissues were obtained from patients diagnosed with HCC *via* histopathological examination. Paired tumor tissues and adjacent non-cancerous samples were obtained at the same time. HCC diagnosis is depending on three factors, namely, chronic liver disease background, the positive iconography examination results, or the positive pathological examination. All HCC patients were free from other viral infections, such as Human Immunodeficiency Virus (HIV), hepatitis virus. These patients were also free from any other types of liver disease (32). All samples were stored at −80°C until further experimental use.

### Cell Lines

Huh-7 cell line (3111C0001CCC000679) was obtained from China Center for Type Culture Collection (CCTCC; Beijing, China) and Dulbecco’s Modified Eagle’s Medium (DME-H-21 4.5 g/Liter Glucose; Invitrogen) supplemented with 10% FBS (Invitrogen). HCCLM3 (C6303) was obtained from Beyotime (Shanghai, China) and cultured in Dulbecco’s Modified Eagle’s Medium (DMEM) supplemented with 10% FBS (Invitrogen).

The human liver cancer cell line Huh-7 and HCCLM3 were exposed to gradually increased concentrations of recombinant human TRAIL (rhTRAIL; 1, 10, 100, and 1,000 ng/ml) as described previously (26, 33, 34) for establishing the TRAIL-resistant Huh-7 (R) and HCCLM3 (R).

### Real-Time qPCR

The target cell monolayers in 12-well plate were rinsed with PBS and harvested for RNA isolation and qPCR as described previously (35). Total RNA (1 μg) was reverse transcribed into cDNA using High-Capacity RNA-to-cDNA Reverse Transcription Kit (Applied Biosystems). The expression levels of lncRNA, miRNA, and mRNA were quantified using an ABI 7500 Fast real time PCR (Applied Biosystems, Thermo Fisher Scientific). The relative mRNA expression was calculated using the 2^−ΔΔCt^ method with β-actin or U6 as an internal reference gene. The primers were listed in **Table S1**.

### Cell Transfection

The overexpression or silencing of lncRNA CASC2 was achieved in cells by transfecting pcDNA3.1-CASC2 (CASC2, vector as a negative control) or vector containing short hairpin RNA targeting CASC2 (sh-CASC2, sh-NC as a negative control). miRNA overexpression or inhibition was achieved in cells by transfecting miRNA mimics or inhibitor (NC mimics or NC inhibitor as a negative control). The overexpression or knockdown of RIPK1 was achieved in cells by transfecting pcDNA3.1-RIPK1 (RIPK1, vector as a negative control) or vector containing short hairpin RNA targeting RIPK1 (sh-RIPK1, sh-NC as a negative control). The primers for vector construction are listed in **Table S1**. All the transfections were performed using Lipofectamine 3000 Reagent (Thermo Fisher Scientific, Waltham, MA, USA).

### MTT Assay for Cell Viability

MTT (3-[4,5-Dimethylthiazole-2-yl]-2,5-diphenyltetrazolium ammonium bromide) was used to detect the cell survival rate according to the method before (36). Cells took in the MTT through the plasma membrane potential, and the MTT was then reduced to formazan by intracellular NAD(P)H-oxidoreductases. Next, the supernatant was discarded, and the DMSO was added for dissolving the formed formazan. Finally, 490 nm OD values were measured and the cell survival rate (cell viability) was calculated taking the non-treated cell (control) viability as 100%.

### EdU Assay for DNA Synthesis Capacity

EdU assay was performed using the Click-IT EdU Alexa Fluor 647 kit (Thermo Fisher, Waltham, MA, USA) as previously described (37, 38). Apollo staining and DAPI staining for nuclei staining were performed. Under a fluorescence microscope, the blue fluorescence represents the nucleus stained by DAPI, and the red fluorescence represents the newly synthesized DNA stained by EdU. The incorporation rate of EdU was equal to the ratio of EdU-positive cells (red)/DAPI-positive cells (blue).

### Flow Cytometry for Cell Apoptosis

Flow cytometry was used to detect apoptosis as previously described (39). Collect cells after digestion by 0.25% trypsin and resuspend with 100 μl of binding buffer. Then, cells were added with 5 μl of Annexin V-FITC and 5 μl of Propidium Iodide (PI) and incubated at room temperature in the dark for 15 min. At the end of the incubation, cell apoptosis was examined by an FACS Calibur FCM (BD Biosciences, San Jose, USA). Experiments in triplicate helped to reduce errors. FACS Diva software was adopted at data analysis.

### Immunoblotting for Protein Levels

The protein levels of cyclin D1, ki67, caspase-8, caspase-3, cleaved-caspase-8, cleaved-caspase-3, RIPK1, IKKβ, p-IκBα, and p-p65 were examined by immunoblotting following the methods described before (36) with antibodies against cyclin D1 (60186-1-Ig; Proteintech, Wuhan, China), ki67 (27309-1-AP, Proteintech), caspase3 (19677-1-AP, Proteintech), cleaved-caspase 3 (ab2302, Abcam, Cambridge, MA, USA), caspase-8 (ab32397, Abcam), cleaved-caspase-8 (# 9496S; Cell Signaling; Danvers, MA, USA), IKKβ (07-1008; Sigma-Aldrich, St. Louis, MO, USA), p-IκBα (#9246; CST, Danvers, MA, USA), p-p65 (ab194726, Abcam), and β-actin (60008-1-Ig, Proteintech). β-actin was taken as an endogenous control. The incubation of membranes with primary antibody was followed by another incubation with HRP-conjugated secondary antibodies. Protein blots were then visualized using enhanced chemilumescent (ECL) substrates (Millipore, MA, USA).

### Lentivirus and Cell Transduction

Human lncRNA CASC2 overexpressing lentivirus (lv-CASC2 TU = 1.5×10^9^/ml) or CASC2 knockdown lentivirus (lv-sh-CASC2 TU = 1.5×10^9^/ml) and negative control lv-NC or lv-sh-NC were prepared by Genechem (Shanghai, China). Human hepatocellular carcinoma cell lines Huh-7 and HCCLM3 were plated into 12-well plates at a density of 4×10^4^ cells/well and then infected with lv-CASC2, lv-sh-CASC2, lv-NC, or lv-sh-NC in serum-free medium, using polybrene (5 µg/µl) reagent to increase the efficiency of infection according to the manufacturer’s protocol. After 12 h incubation, the medium was changed to DMEM supplemented with 10% FBS. Then, the cells were incubated for another 48 h before proceeding with experiments.

### Subcutaneous Xenotransplant Tumor Model in Nude Mice

Huh-7 cells infected with lentivirus overexpressing lncRNA CASC2 or negative control lentivirus were implanted subcutaneously to the left flank of female nude mice (BALB/c mice; 18–22 g, 5 weeks old, obtained from SLAC laboratory animal center, Changsha, China). Tumor volume was measured every 3 days from the 9th day after injection, when the tumor began to form. On the 25th day, nude mice were sacrificed and the tumor weight was measured. Tumor tissues were collected to detect the content of related proteins.

### Hematoxylin and Eosin Staining for Histological Analysis

Tumor tissues were weighed and cut immediately after sacrificing the mice; further, the tumor tissues were fixed in 10% neutral-buffered formalin overnight. The sections were stained with hematoxylin and eosin (H&E) to observe the morphological changes.

### Dual-Luciferase Reporter Assay

For validating miR-18a binding RIPK1 3’-UTR and lncRNA CASC2, respectively, and RELA binding CASC2 promoter region, dual-luciferase reporter assay was performed. CASC2 or RIPK1 3’-UTR was amplified by PCR using genomic DNA of the Huh-7 cell line and cloned downstream of the Renilla luciferase open reading frame in the Renilla psiCHECK2 vector (Promega, Madison, WI, USA). Mutations were introduced to the seed region of the miR-18a binding site in CASC2 or RIPK1 3’-UTR, and the construct was named mut-CASC2 or mut-RIPK1 3’-UTR. Next, 293T cells were seeded in 96-well plates and co-transfected with miR-18a-5p mimics/ miR-18a-5p inhibitor together with psiCHECK-2 reporter vectors (wt-/mut-RIPK1 3’-UTR or wt-/mut-CASC2). The luciferase activity was measured using the dual-luciferase reporter gene detection system (Promega) according to the manufacturer’s instructions 48 h following the transfection. The values are double-normalized to cells transfected with blank psiCHECK-2 control vector and firefly luciferase activity.

### Chromatin Immunoprecipitation-PCR Analysis

ChIP was performed with anti-IgG or anti-NF-κB. Briefly, cells at a concentration of 2×10^6^ cells/ml were treated with 1% formaldehyde in medium for 10 min at room temperature. After two washes with ice-cold PBS containing protease inhibitors, the cells were pelleted by centrifugation and resuspended in SDS lysis buffer. After incubation for 15 min at 4°C, the lysates were sonicated 12 times (30 s each). After centrifugation, the supernatant was diluted in ChIP dilution buffer and incubated overnight at 4°C with anti-IgG or anti-NF-κB and protein G beads. Samples were washed two times in lysis buffer, four times in 1 M lysis buffer (50 mM Tris, pH 7.4, 1 M NaCl, 1 mM EDTA, 0.1% SDS, 1% NP-40, and 0.5% sodium deoxycholate), and the beads were then resuspended in lysis buffer and treated with proteinase K at 45°C for 45 min. Coprecipitated DNAs were purified using a QIAquick DNA purification spin column (Qiagen, Germantown, MD, USA) and eluted in 50 μl nuclease-free water. The immunoprecipitated DNA was quantified using PCR, and all values were normalized to the input.

### Statistical Analysis

All the experiments were repeated for at least three times. Data from at least three independent experiments are processed by GraphPad (GraphPad Software, San Diego, CA, USA) and then presented as the mean ± S.D. Where applicable, the Student’s *t*-test is used for statistical comparison between means. In the above analysis, the difference between more than two groups was estimated using one-way analysis of variance and Turkish *post-hoc* test. A P value of less than 0.05 is considered statistically different.

## Results

### *In Vitro* Effects of lncRNA CASC2 on HCC Cells

**Figure 1:**
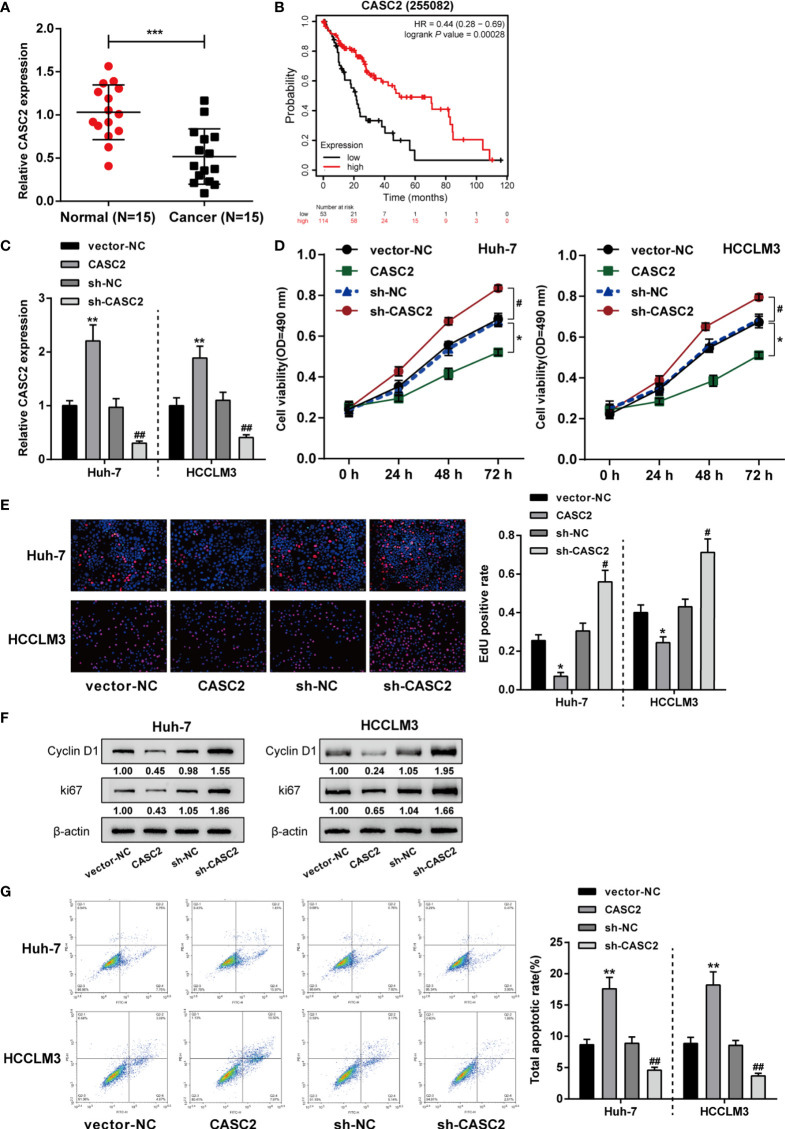
*In vitro* effects of lncRNA CASC2 on hepatocellular carcinoma (HCC) cells. **(A)** LncRNA CASC2 expression was determined in HCC (n=15) and adjacent non-cancerous tissues (n=15) using qRT-PCR. **(B)** The correlation of CASC2 expression and the survival probability of 167 free from hepatitis virus infections HCC patients was analyzed using Kaplan-Meier Plotter online tool (https://kmplot.com/analysis/index.php?p=service) and a log-rank analysis. **(C)** The overexpression or silencing of lncRNA CASC2 was achieved in cells by transfecting pcDNA3.1-CASC2 (CASC2, empty vector as a negative control) or vector short hairpin RNA targeting CASC2 (sh-CASC2, sh-NC as a negative control). The transfection efficiency was confirmed by real-time PCR. Then, HCC cell lines Huh-7 and HCCLM3 were transfected with CASC2 or sh-CASC3 and examined for cell viability by MTT assay **(D)**; DNA synthesis capacity by EdU assay **(E)**; the protein levels of proliferating markers cyclin D1 and ki67 by Immunoblotting **(F)**; cell apoptosis by Flow cytometry assay **(G)**. *p < 0.05, **p < 0.01, ***p < 0.005 compared with normal or vector-NC group, ^#^p < 0.05, ^##^p < 0.01, compared with sh-NC group.

Given the tumor-suppressive role of CASC2 in HCC by our and other groups’ studies (26, 40–42), the expression of CASC2 was first validated in collected clinical tissue samples and cell lines. **Figure 1A** showed that in 15 cases HCC samples, CASC2 expression was significantly downregulated compared with that in normal non-cancerous samples. More importantly, Kaplan-Meier Plotter online tool (https://kmplot.com/analysis/index.php?p=service) indicated that higher CASC2 expression was a protective factor for HCC patients (**Figure 1B**). To validate the specific effects of CASC2, CASC2 overexpression or silencing was achieved in Huh-7 and HCCLM3 cells by transfecting CASC2-overexpressing vector [CASC2, empty vector (vector-NC) as a negative control] or vector containing short hairpin RNA against CASC2 (sh-CASC2, sh-NC as a negative control), and employed real-time PCR to verify the transfection efficiency and CASC2 overexpressing or silencing in HCC cells was successfully conducted (**Figure 1C**). Then, Huh-7 and HCCLM3 cells were transfected accordingly and examined for cell phenotypes. **Figures 1D, E** showed that CASC2 overexpression markedly inhibited, whereas CASC2 silencing promoted cell viability and DNA synthesis capacity. Consistently, CASC2 overexpression significantly downregulated, whereas CASC2 silencing upregulated the protein contents of proliferating markers cyclin D1 and ki67 (**Figure 1F**). As shown in **Figure 1G**, CASC2 overexpression promoted, whereas CASC2 silencing repressed cell apoptosis.

### *In Vivo* Effects of lncRNA CASC2 on Huh-7 Cell Tumor Growth

**Figure 2:**
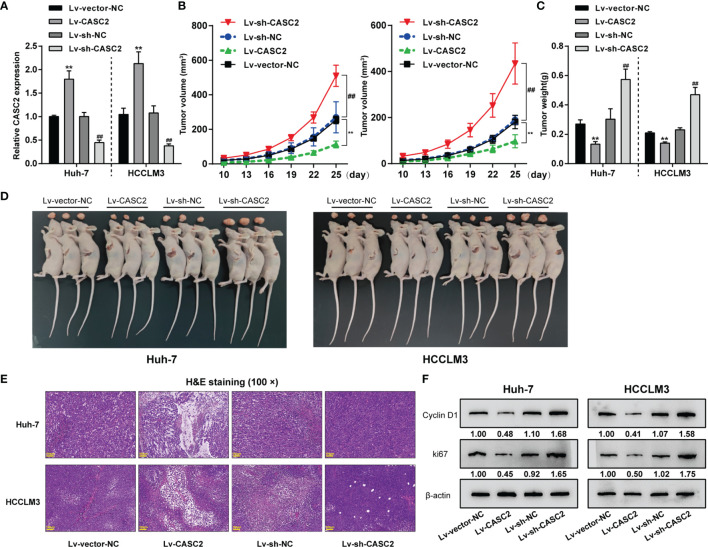
*In vivo* effects of lncRNA CASC2 on Huh-7 cell tumor growth. **(A)** Subcutaneous xenotransplant tumor model was established in nude mice by injecting Huh-7 or HCCLM3 cells infected with human lncRNA CASC2-overexpressing lentivirus (lv-CASC2) or negative control lv-NC and CASC2-knockdown lentivirus (lv-sh-CASC2) or corresponding control (lv-sh-NC). The expression of lncRNA CASC2 in Huh-7 or HCCLM3 cells were examined by real-time qPCR. **(B)** Tumor volume was measured every 3 days from the 10th day after injection, when the tumor began to form. **(C)** On the 25th day, nude mice were sacrificed and the tumor weight was measured. **(D)** Images of the tumors in each group. **(E)** The histopathological characteristics of the tumors were examined by hematoxylin and eosin (H&E) staining. **(F)** The protein levels of proliferating markers in tumors were examined by Immunoblotting. **p < 0.01, compared with lv-vector-NC, ^##^p < 0.01 compared with Lv-sh-NC group.

To verify the *in vitro* findings, subcutaneous xenotransplant tumor model was established in nude mice by injecting Huh-7 or HCCLM3 cells infected with human lncRNA CASC2-overexpressing lentivirus (lv-CASC2) or negative control lv-NC and CASC2-knockdown lentivirus (lv-sh-CASC2) or shRNA control lv-sh-NC. lncRNA CASC2 overexpression or knockdown within Huh-7 or HCCLM3 cells were examined by real-time qPCR (**Figure 2A**). Tumor volume was measured every 3 days from day 10; **Figure 2B** showed that CASC2 overexpression in Huh-7 or HCCLM3 cells dramatically decreased tumor volumes compared with the lv-NC group; while CASC2 silencing notably promoted tumor volumes. On the 25th day, nude mice were sacrificed and the tumor weight was measured; consistently, CASC2 overexpression in Huh-7 or HCCLM3 cells considerably decreased the tumor weight as compared to the lv-NC group; while CASC2 silencing showed the opposite effect (**Figure 2C**). Images of the tumors in each group are shown in **Figure 2D**. H&E staining was then performed to examine the histopathological characteristics of the tumors; **Figure 2E** shows that CASC2 overexpression in Huh-7 or HCCLM3 cells improved the necrosis of tumor tissues; while CASC2 silencing markedly inhibited necrosis of tumor tissues. Then, the protein levels of proliferating and apoptosis markers in tumor tissues were also examined. Consistently, CASC2 overexpression significantly decreased and CASC2 silencing increased proliferating markers cyclin D1 and ki67 in tumor tissues (**Figure 2F**). Thus, CASC2 overexpression might repress subcutaneously transplanted tumor growth in mice; while knockdown of CASC2 efficiently facilitated the tumorigenesis ability of HCC cells.

### Effects of lncRNA CASC2 on Caspase-Cascades and the NF-κB Pathway

**Figure 3:**
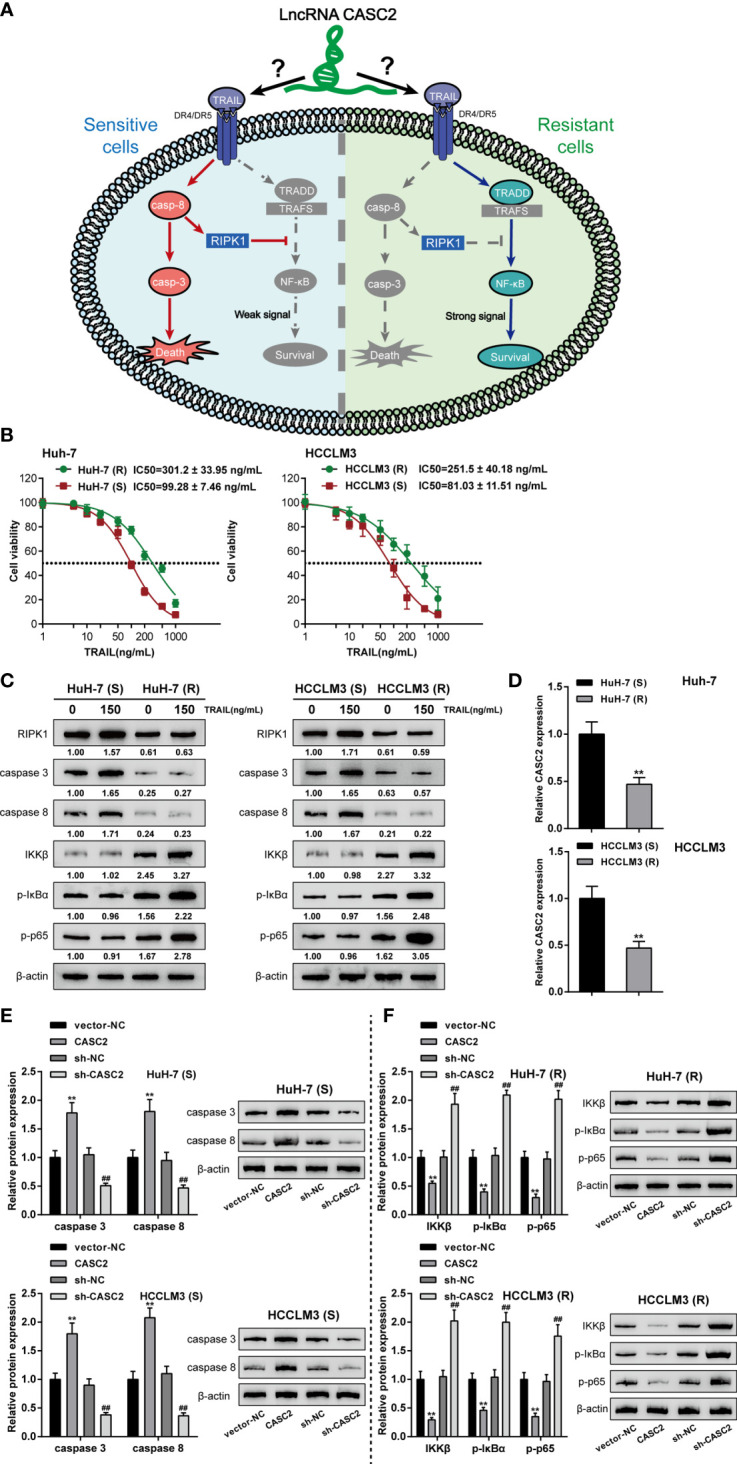
Effects of lncRNA CASC2 on caspase-cascades and the NF-κB pathway. **(A)** A schematic diagram showing two different modes of TRAIL acting on TRAIL-sensitive and TRAIL-resistant cell lines. **(B)** Huh-7 and HCCLM3 cells were exposed to 1, 10, 100, and 1,000 ng/ml rhTRAIL protein and examined for the cell viability by MTT assay. The IC50 values were calculated and shown. Regular Huh-7 and HCCLM3 cells were then divided into TRAIL-sensitive Huh-7 (S) and HCCLM3 (S) and TRAIL-resistant Huh-7 (R) and HCCLM3 (R). **(C)** Huh-7 (S), HCCLM3 (S), Huh-7 (R), and HCCLM3 (R) cells were treated with 0 or 150 ng/ml rhTRAIL and examined for the protein levels of RIPK1, caspase-8, caspase-3, IKKβ, p-IκBα, and p-p65 using Immunoblotting. **(D)** CASC2 expression was determined in Huh-7 (S), HCCLM3 (S), Huh-7 (R), and HCCLM3 (R) cells using qRT-PCR. **(E)** Huh-7 (S) and HCCLM3 (S) cells were transfected with CASC2 or sh-CASC2 and examined for the protein levels of caspase-8 and caspase-3 using Immunoblotting. **(F)** Huh-7 (R) and HCCLM3 (R) cells were transfected with CASC2 or sh-CASC2 and examined for the protein levels of IKKβ, p-IκBα, and p-p65 using Immunoblotting. **p < 0.01, compared with sensitive group or vector-NC group, ^##^p < 0.01, compared with sh-NC group.

TRAIL has two different modes of action in TRAIL-sensitive and TRAIL-resistant cell lines as in previous studies (43) (**Figure 3A**). Therefore, it is reasonable to speculate that lncRNA CASC2 affects the response of cancer cells to TRAIL through two different modes of action. To select proper cell model for investigation, regular and TRAIL-resistant Huh-7 [Huh-7 (R) and Huh-7 (S)] and HCCLM3 cells [HCCLM3 (R) and HCCLM3 (S)] were treated with 1, 10, 100, or 1,000 ng/ml TRAIL, the cell viability was determined and shown as IC50 values. **Figure 3B** showed that compared with the Huh-7 (S) and HCCLM3 (S) cells, IC50 values for Huh-7 (R) and HCCLM3 (R) were significantly elevated (P value = 0.0005 or 0.0021, respectively).

Next, to monitor the alterations of the caspase cascades and the NF-κB signaling, regular and TRAIL-resistant cells were treated with 0 or 150 ng/ml TRAIL and examined for the protein levels of caspase cascades key factors (RIPK1, caspase-8, and caspase-3) and NF-κB signaling key factors (IKKβ, p-IκBα, and p-p65). **Figure 3C** showed that the protein levels of caspase cascades factors, RIPK1, caspase-8, and caspase-3, in TRAIL-resistant Huh-7 (R) and HCCLM3 (R) cells were reduced compared to those in the TRAIL-sensitive Huh-7 (S) and HCCLM3 (S) cells; on the contrary, NF-κB signaling key factors, IKKβ, p-IκBα, and p-p65 proteins, were notably increased in Huh-7 (R) and HCCLM3 (R) cells when compared to Huh-7 (S) and HCCLM3 (S) cells, suggesting the suppression of caspase cascades and the activation of the NF-κB signaling were observed in TRAIL-treated resistant cells when compared to TRAIL-treated sensitive cells. Besides, the proteins of RIPK1, caspase-8, and caspase-3 were significantly increased in 150 ng/ml TRAIL-treated sensitive Huh-7 (S) and HCCLM3 (S) cells when compared to 0 ng/ml TRAIL-treated Huh-7 (S) and HCCLM3 (S) cells; contrariwise, IKKβ, p-IκBα, and p-p65 proteins were significantly increased in 150 ng/ml TRAIL-treated resistant Huh-7 (R) and HCCLM3 (R) cells when compared to 0 ng/ml TRAIL-treated Huh-7 (R) and HCCLM3 (R) cells, suggesting the activation of caspase cascades and the NF-κB signaling in TRAIL-treated sensitive or resistant cell lines under TRAIL treatment. In the meantime, lncRNA CASC2 expression was significantly downregulated in Huh-7 (R) and HCCLM3 (R) cells compared with Huh-7 (S) and HCCLM3 (S) cells (**Figure 3D**), further suggesting that lncRNA CASC2 might affect the response of cancer cells to TRAIL through different mechanisms.

Considering the activation of the caspase cascades in TRAIL-sensitive cell lines, CASC2 overexpression or knockdown was achieved in Huh-7 (S) and HCCLM3 (S) cells, and the levels of caspase-8 and caspase-3 were examined. **Figure 3E** showed that in Huh-7 (S) and HCCLM3 (S) cells, CASC2 overexpression increased, whereas CASC2 knockdown decreased the levels of caspase-8 and caspase-3. Considering the activation of the NF-κB signaling in TRAIL-resistant cell lines, CASC2 overexpression or knockdown was achieved in Huh-7 (R) and HCCLM3 (R) cells, and the levels of IKKβ, p-IκBα, and p-p65 were examined. **Figure 3F** showed that in Huh-7 (R) and HCCLM3 (R) cells, CASC2 overexpression decreased, whereas CASC2 knockdown increased the levels of IKKβ, p-IκBα, and p-p65. These data indicate that CASC2 indeed affects the activation of the caspase cascades and the NF-κB signaling in TRAIL-sensitive or TRAIL-resistant cell lines in different ways.

### LncRNA CASC2 Regulates Caspase-8 and Caspase-3 by Targeting miR-24/221

**Figure 4:**
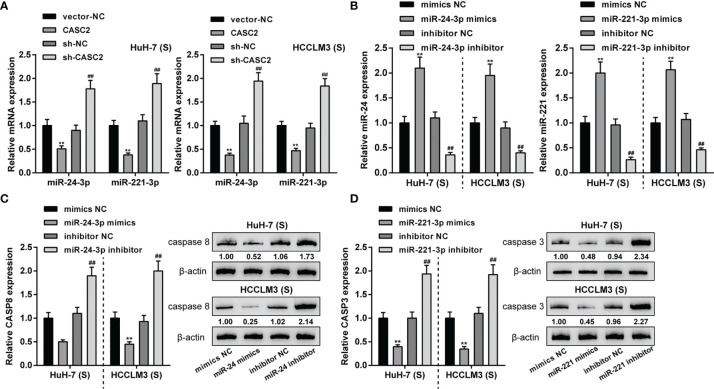
LncRNA CASC2 regulates caspase-8 and caspase-3 by targeting miR-24/221. **(A)** Huh-7 (S) and HCCLM3 (S) cells were transfected with CASC2 or sh-CASC2 and examined for the expression of miR-24 and miR-221 expression using qRT-PCR. **(B)** miR-24/miR-221 overexpression or inhibition was achieved in Huh-7 (S) and HCCLM3 (S) cells by transfecting miR-24/miR-221 mimics or inhibitor; transfection efficiency was confirmed using qRT-PCR. **(C)** Huh-7 (S) and HCCLM3 (S) cells were transfected with miR-24 mimics or inhibitor and examined for caspase-8 protein using Immunoblotting. **(D)** Huh-7 (S) and HCCLM3 (S) cells were transfected with miR-221 mimics or inhibitor and examined for caspase-3 protein using Immunoblotting. **p < 0.01, compared with vector-NC or mimics NC group, ^##^p < 0.01, compared with sh-NC or inhibitor NC group.

In our previous study, we demonstrated CASC2/miR-24/caspase-8 and CASC2/miR-221/caspase-3 axes in HCC cells, modulating HCC cell resistance to TRAIL (26). Thus, CASC2/miR-24/caspase-8 and CASC2/miR-221/caspase-3 axes might be the mechanism of the caspase cascades activation in Huh-7 (S) and HCCLM3 (S) cells. In Huh-7 (S) and HCCLM3 (S) cells, CASC2 overexpression downregulated, whereas CASC2 knockdown upregulated miR-24 and miR-221 expression (**Figure 4A**). Next, miR-24/miR-221 overexpression or inhibition was achieved in Huh-7 (S) and HCCLM3 (S) cells by transfecting miR-24/miR-221 mimics or inhibitor (**Figure 4B**). In Huh-7 (S) and HCCLM3 (S) cells, miR-24 overexpression decreased, whereas miR-24 inhibition increased caspase-8 protein (**Figure 4C**). In Huh-7 (S) and HCCLM3 (S) cells, miR-221 overexpression decreased, whereas miR-221 inhibition increased caspase-3 protein (**Figure 4D**). Thus, in Huh-7 (S) and HCCLM3 (S) cells, CASC2 modulates the activation of the caspase cascades through the miR-24/caspase-8 and miR-221/caspase-3 axes.

### CASC2/miR-24/Caspase-8 and CASC2/miR-221/Caspase-3 Axes Modulate Apoptosis in Huh-7 (S) and HCCLM3 (S) Cells

**Figure 5:**
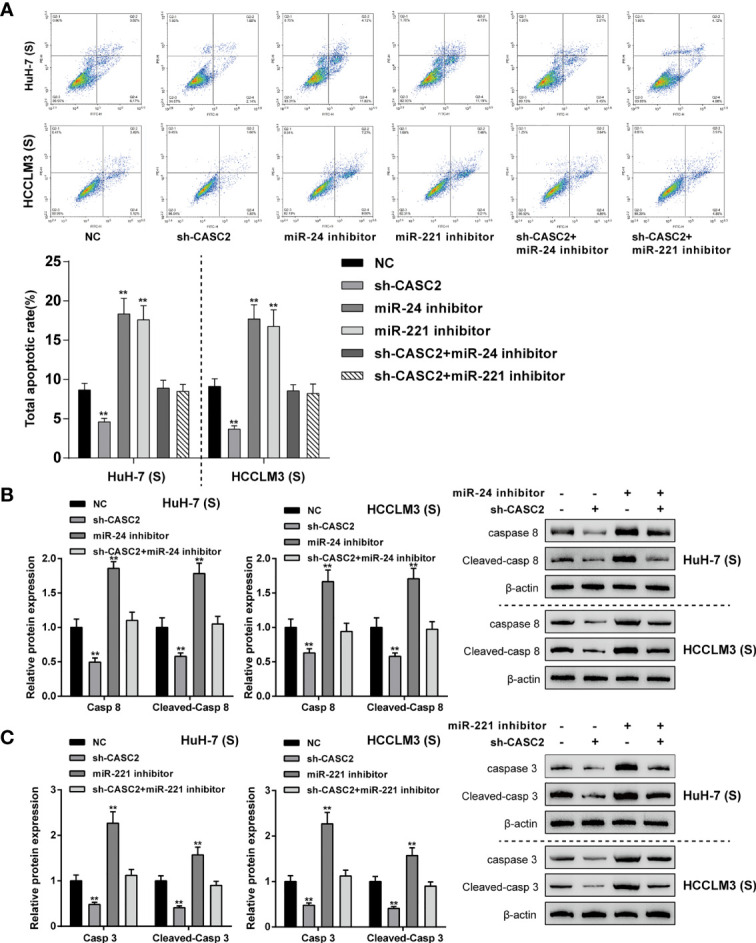
CASC2/miR-24/caspase-8 and CASC2/miR-221/caspase-3 axes modulate apoptosis in Huh-7 (S) and HCCLM3 (S) cells. Huh-7 (S) and HCCLM3 (S) cells were divided into six groups: NC, sh-CASC2, miR-24 inhibitor, miR-221 inhibitor, sh-CASC2+miR-24 inhibitor, and sh-CASC2+miR-221 inhibitor; Huh-7 (S) and HCCLM3 (S) cells were transfected accordingly and examined for cell apoptosis using Flow cytometry **(A)**; the protein levels of caspase-8 and cleaved-caspase-8 in NC, sh-CASC2, miR-24 inhibitor, and sh-CASC2+miR-24 inhibitor groups using Immunoblotting **(B)**; the protein levels of caspase-3 and cleaved-caspase-3 in NC, sh-CASC2, miR-221 inhibitor, and sh-CASC2+miR-221 inhibitor groups using Immunoblotting **(C)**. **p < 0.01 compared with NC group.

Given that CASC2 modulates the activation of the caspase cascades through the miR-24/caspase-8 and miR-221/caspase-3 axes in Huh-7 (S) and HCCLM3 (S) cells, next, the role of these two axes in Huh-7 (S) and HCCLM3 (S) cell apoptosis was investigated. Firstly, Huh-7 (S) and HCCLM3 (S) cells were divided into six groups: NC, sh-CASC2, miR-24 inhibitor, miR-221 inhibitor, sh-CASC2+miR-24 inhibitor, and sh-CASC2+miR-221 inhibitor; Huh-7 (S) and HCCLM3 (S) cells were transfected accordingly and examined for cell apoptosis. In both cell lines, CASC2 knockdown significantly inhibited, whereas miR-24 inhibition or miR-221 inhibition promoted cell apoptosis (**Figure 5A**); when co-transfected with sh-CASC2 and miR-24 inhibitor or sh-CASC2 and miR-221 inhibitor, the effects of CASC2 knockdown were significantly attenuated by miR-24 inhibition or miR-221 inhibition (**Figure 5A**). Then, the protein levels of caspase-8 and cleaved-caspase-8 were examined in NC, sh-CASC2, miR-24 inhibitor, and sh-CASC2+miR-24 inhibitor groups, whereas caspase-3 and cleaved-caspase-3 were examined in NC, sh-CASC2, miR-221 inhibitor, and sh-CASC2+miR-221 inhibitor groups. **Figure 5B** showed that CASC2 knockdown significantly decreased, whereas miR-24 inhibition increased the levels of caspase-8 and cleaved-caspase-8; the effects of CASC2 knockdown could be significantly attenuated by miR-24 inhibition. Similarly, **Figure 5C** showed that CASC2 knockdown significantly decreased, whereas miR-221 inhibition increased the levels of caspase-3 and cleaved-caspase-3; the effects of CASC2 knockdown could be significantly attenuated by miR-221 inhibition.

### LncRNA CASC2 Targets miR-18a to Modulate miR-18a Downstream RIPK1

**Figure 6:**
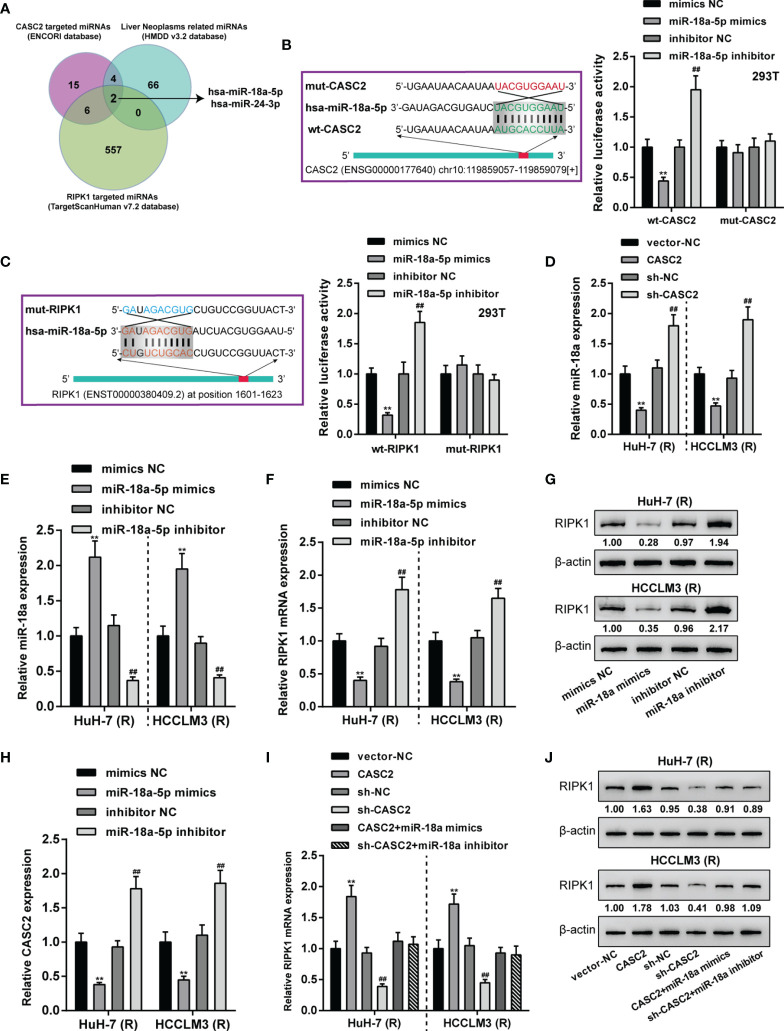
LncRNA CASC2 targets miR-18a to modulates miR-18a downstream RIPK1. **(A)** ENCORI and TargetScan 7.2 were used to analyze miRNAs that might bind to CASC2 and RIPK1; HMDD v3.2 data were used to analyze miRNAs that might relate to liver neoplasms. miRNAs obtained from three tools intersected in miR-18a and miR-24-3p. **(B, C)** Wild- and mutant-type CASC2 and RIPK1 luciferase reporter plasmids were constructed. The predicted miR-18a binding sites in mutant-type CASC2 and RIPK1 reporter plasmids were mutated. These reporter plasmids were then co-transfected in 293T cells with miR-18a mimics or inhibitor, and the luciferase activity was determined. **(D)** Huh-7 (R) and HCCLM3 (R) cells were transfected with CASC2 or sh-CASC2 and examined for miR-18a expression using qRT-PCR. **(E)** miR-18a overexpression or inhibition was achieved in Huh-7 (R) and HCCLM3 (R) cells by transfecting miR-18a mimics or inhibitor; miR-18a expression was confirmed using qRT-PCR. **(F–H)** Huh-7 (R) and HCCLM3 (R) cells were transfected with miR-18a mimics or inhibitor and examined for RIPK1 mRNA **(F)** and protein **(G)** levels or CASC2 mRNA **(H)** expression using RT-PCR and Immunoblotting. **(I, J)** Huh-7 (R) and HCCLM3 (R) cells were transfected with CASC2 or sh-CASC2 and miR-18a mimics or inhibitor and examined for RIPK1 mRNA **(I)** and protein **(J)** levels using RT-PCR and Immunoblotting. **p < 0.01, compared with vector-NC or mimics NC group, ^##^p < 0.01, compared with sh-NC or inhibitor NC group.

Given that RIPK1 was significantly downregulated in Huh-7 (R) and HCCLM3 (R) cells, next, ENCORI and TargetScan 7.2 were used to analyze miRNAs that might bind to CASC2 and RIPK1; moreover, HMDD v3.2 data were used to analyze miRNAs that might relate to liver neoplasms. **Figure 6A** showed that miRNAs obtained from three tools intersected in miR-18a and miR-24-3p. In our previous study (26), we have confirmed the bind relationship between miR-24-3p and CASC2. Here we investigated the predicted bindings between CASC2 and miR-18a or miR-18a and RIPK1 using dual-luciferase reporter assay. Wild- and mutant-type CASC2 and RIPK1 luciferase reporter plasmids were constructed. The predicted miR-18a binding sites in mutant-type CASC2 and RIPK1 reporter plasmids were mutated. These reporter plasmids were then co-transfected in 293T cells with miR-18a mimics or inhibitor. **Figures 6B, C** showed that when co-transfected with wild-type CASC2 or RIPK1 reporter plasmid, miR-18a overexpression inhibited, whereas miR-18a inhibition enhanced the luciferase activity of wt-CASC2 or wt-RIPK1; when co-transfected with mutant-type CASC2 or RIPK1 reporter plasmid, miR-18a overexpression or inhibition failed to alter the luciferase activity. Thus, CASC2 directly binds to miR-18a and miR-18a directly binds to RIPK1. In Huh-7 (R) and HCCLM3 (R) cells, CASC2 overexpression downregulated, whereas CASC2 inhibition upregulated miR-18a expression (**Figure 6D**). In Huh-7 (R) and HCCLM3 (R) cells, miR-18a overexpression or inhibition was achieved by transfecting miR-18a mimics or inhibitor (**Figure 6E**); miR-18a overexpression downregulated RIPK1 mRNA and decreased RIPK1 protein, whereas miR-18a inhibition exerted opposite effects on RIPK1 mRNA and protein (**Figures 6F, G**). Besides, miR-18a overexpression notably restrained CASC2 expression, whereas miR-18a inhibition promoted CASC2 expression in Huh-7 (R) and HCCLM3 (R) cells (**Figure 6H**). Moreover, in Huh-7 (R) and HCCLM3 (R) cells, CASC2 overexpression upregulated RIPK1 mRNA and increased RIPK1 protein, whereas CASC2 knockdown exerted opposite effects; the effect of CASC2 on RIPK1 mRNA and protein expression could be offset by miR-18a (**Figures 6I, J**). Thus, CASC2 acts as a competing endogenous RNA (ceRNA) for miR-18a and counteracts miR-18a-mediated RIPK1 suppression.

### Dynamic Effects of the CASC2/miR-18a/RIPK1 Axis on TRAIL-Resistant HCC Cell Proliferation

**Figure 7:**
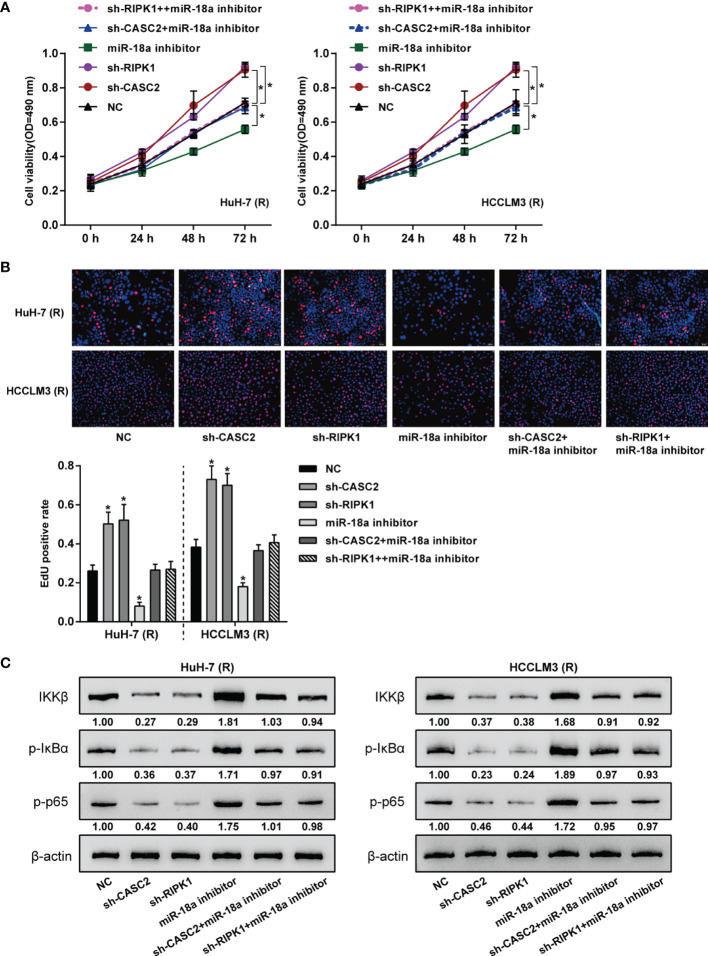
Dynamic effects of the CASC2/miR-18a/RIPK1 axis on TRAIL-resistant HCC cell proliferation. Huh-7 (R) and HCCLM3 (R) cells were divided into six groups: NC, sh-CASC2, sh-RIPK1, miR-18a inhibitor, sh-CASC2+ miR-18a inhibitor, and sh-RIPK1+ miR-18a inhibitor; cells in different groups were transfected accordingly and examined for cell viability using CCK-8 assay **(A)**; DNA synthesusing EdU assay **(B)**; the protein levels of IKKβ, p-IκBα, and p-p65 using Immunoblotting **(C)**. *p < 0.05 compared with NC group.

After confirming the CASC2/miR-18a/RIPK1 axis in TRAIL-resistant Huh-7 (R) and HCCLM3 (R) cells, the dynamic effects of the axis on Huh-7 (R) and HCCLM3 (R) cell phenotypes were investigated. Huh-7 (R) and HCCLM3 (R) cells were divided into six groups: NC, sh-CASC2, sh-RIPK1, miR-18a inhibitor, sh-CASC2+ miR-18a inhibitor, and sh-RIPK1+ miR-18a inhibitor; cells in different groups were transfected accordingly and examined for cell viability and DNA synthesis. **Figures 7A, B** showed that miR-18a inhibition inhibited, whereas CASC2 knockdown or RIPK1 knockdown promoted Huh-7 (R) and HCCLM3 (R) cell viability and DNA synthesis; when co-transfected, the effects of CASC2 knockdown were partially attenuated by miR-18a inhibition, whereas miR-18a inhibition effects were partially attenuated by RIPK1 knockdown. Consistently, miR-18a inhibition increased, whereas CASC2 knockdown or RIPK1 knockdown decreased the protein levels of IKKβ, p-IκBα, and p-p65 in Huh-7 (R) and HCCLM3 (R) cells; when co-transfected, the effects of CASC2 knockdown on these proteins were partially attenuated by miR-18a inhibition, whereas miR-18a inhibition effects on these proteins were partially attenuated by RIPK1 knockdown (**Figure 7C**).

### The Transcription Factor NF-κB Inhibits lncRNA CASC2 Transcription

**Figure 8:**
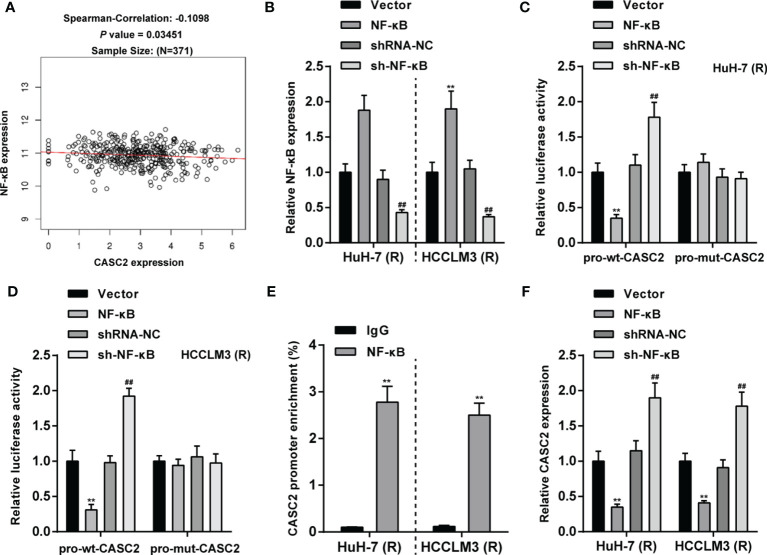
The transcription factor NF-κB inhibits lncRNA CASC2 transcription. **(A)** TCGA hepatocellular carcinoma data (TCGA_LIHC) were analyzed using Spearman’s correlation analysis for the correlation between lncRNA CASC2 and RELA. **(B)** NF-κB overexpression or knockdown was achieved in Huh-7 (R) and HCCLM3 (R) cells by transfecting NF-κB-overexpressing plasmid (NF-κB) or short hairpin RNA targeting NF-κB (sh-NF-κB); the transfection efficiency was confirmed using qRT-PCR. **(C, D)** Wild- and mutant-type CASC2 promoter luciferase reporter plasmids were co-transfected with NF-κB or sh-NF-κB, and the luciferase activity was determined. **(E)** ChIP assay was performed using anti-IgG or anti-NF-κB, and the levels of CASC2 promoter in immunoprecipitate by anti-IgG or anti-NF-κB were determined using qRT-PCR. **(F)** Huh-7 (R) and HCCLM3 (R) cells were transfected with NF-κB or sh-NF-κB and examined for CASC2 expression using qRT-PCR. **p < 0.01, compared with vector or IgG group, ^##^p < 0.01, compared with shRNA-NC group.

The transcription factor binding sites of lncRNA CASC2 gene promoter region in liver cancer tissues were analyzed by ChIP-ATLAS (https://chip-atlas.org/). It was found that lncRNA CASC2 gene had RELA (p65) binding site on both the upstream and downstream 2kb of transcription start site (TSS) in Huh-7 cells (**Table S2**). Then, JASPAR (http://jaspar.genereg.net/) predicted three RELA binding sites with high score (score > 8) in lncRNA CASC2 promoter regions (**Table S3**). TCGA hepatocellular carcinoma data (TCGA_LIHC) were analyzed using Spearman’s correlation analysis, and it has been found that lncRNA CASC2 was weakly negatively correlated with RELA (**Figure 8A**). Then, NF-κB overexpression or knockdown was achieved in Huh-7 (R) and HCCLM3 (R) cells by transfecting NF-κB-overexpressing plasmid (NF-κB) or short hairpin RNA targeting NF-κB (sh-NF-κB); the transfection efficiency was confirmed using qRT-PCR (**Figure 8B**). To confirm the predicted binding, dual-luciferase reporter and ChIP assays were performed. Wild- and mutant-type CASC2 promoter luciferase reporter plasmids were co-transfected with NF-κB or sh-NF-κB. **Figures 8C, D** showed that when co-transfected with pro-wt-CASC2, NF-κB overexpression inhibited, whereas NF-κB inhibition promoted the luciferase activity; when co-transfected with mut-CASC2, NF-κB overexpression or inhibition caused no changes in the luciferase activity. Moreover, ChIP assay showed that compared with immunoprecipitate by anti-IgG, the levels of CASC2 promoter were significantly higher in immunoprecipitate by anti-NF-κB (**Figure 8E**), indicating the direct binding of NF-κB to CASC2 promoter region. In Huh-7 (R) and HCCLM3 (R) cells, NF-κB overexpression downregulated, whereas NF-κB inhibition upregulated CASC2 expression (**Figure 8F**). These data indicate that NF-κB binds to CASC2 promoter region and inhibits CASC2 transcription.

### Expression and Correlation of Aforementioned Factors in Tissue Samples

**Figure 9:**
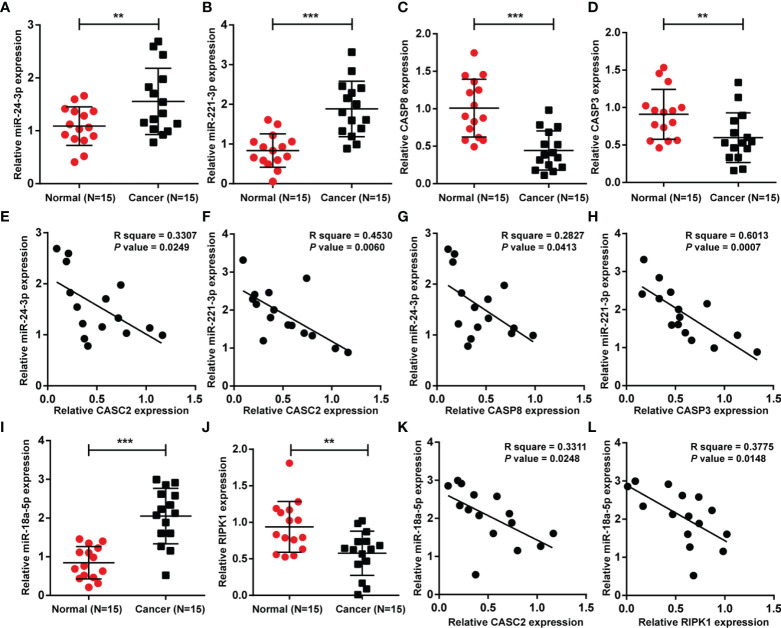
Expression and correlation of aforementioned factors in tissue samples. **(A–D)** The expression of miR-24-3p, miR-221-3p, caspase-8, and caspase-3 was determined in HCC tissues and non-cancerous tissues using qRT-PCR. **(E–H)** The correlations between CASC2, miR-24-3p, miR-221-3p, caspase-8, and caspase-3 were analyzed using Pearson’s correlation analyses. **(I, J)** miR-18a and RIPK1 expression was determined in HCC tissues and non-cancerous tissues using qRT-PCR. **(K, L)** The correlations between CASC2, miR-18a, and RIPK1 were analyzed using Pearson’s correlation analyses. **p < 0.01, ***p < 0.005, compared with normal group.

Finally, the expression of miR-24-3p, miR-221-3p, caspase-8, and caspase-3 was determined in tissue samples; **Figures 9A–D** showed that miR-24-3p and miR-221-3p were significantly upregulated, whereas caspase-8 and caspase-3 were downregulated in HCC tissues compared with non-cancerous tissues. In tissue samples, miR-24-3p was negatively correlated with CASC2 (**Figure 9E**) and caspase-8 (**Figure 9G**), respectively; miR-221-3p was negatively correlated with CASC2 (**Figure 9F**) and caspase-3 (**Figure 9H**), respectively. Moreover, miR-18a expression was significantly upregulated, whereas RIPK1 expression was downregulated in HCC tissues compared with non-cancerous tissues (**Figures 9I, J**). In tissue samples, miR-18a was negatively correlated with CASC2 (**Figure 9K**) and RIPK1 (**Figure 9L**), respectively.

## Discussion

**Figure 10:**
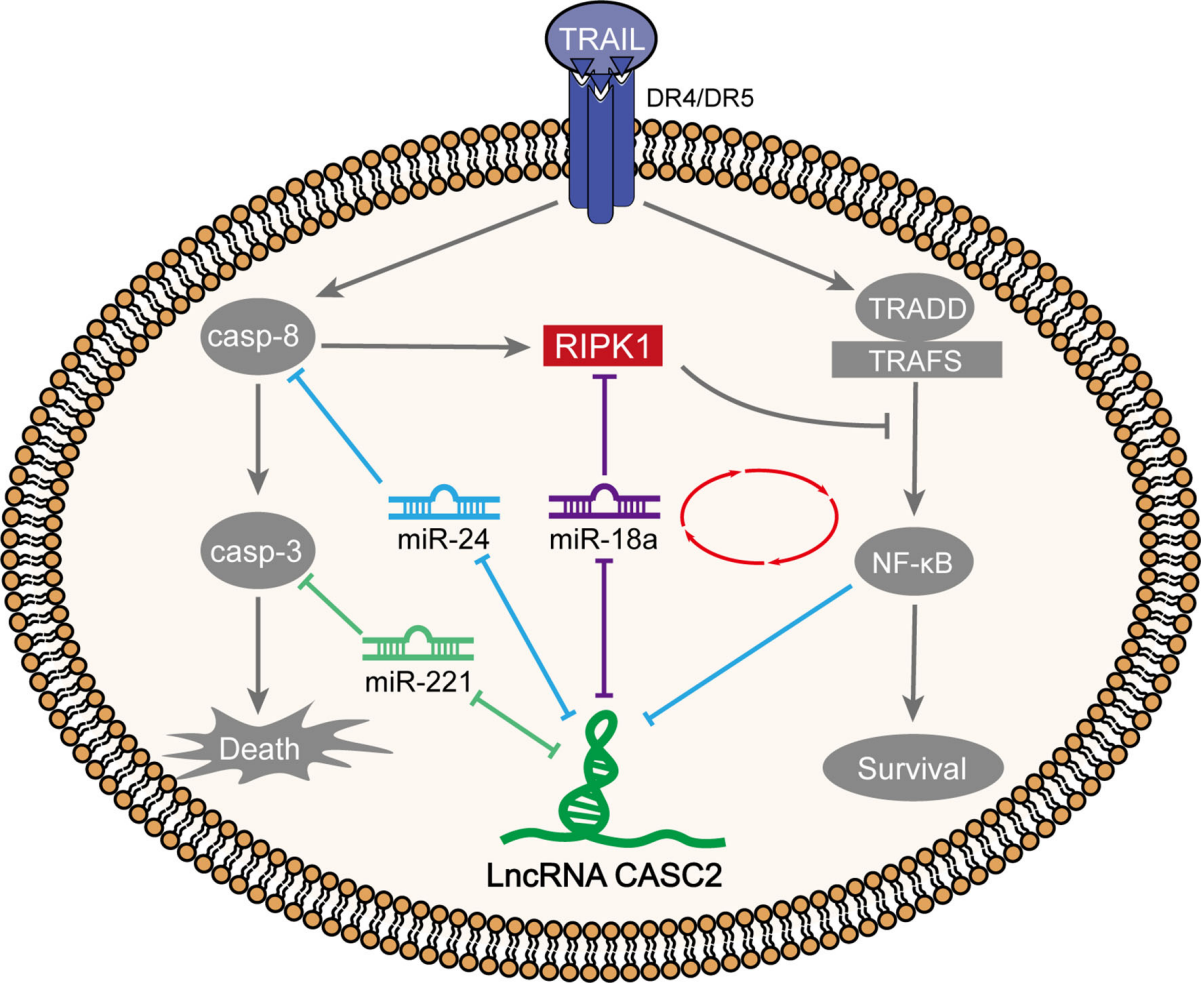
A schematic diagram showing two different modes of TRAIL acting on TRAIL-sensitive and TRAIL-resistant cell lines through the caspase cascades and NF-κB signaling, respectively.

Tumor-suppressive CASC2 (26) was downregulated in HCC tissues and cell lines; HCC patients with lower CASC2 expression predicted a shorter overall survival rate. *In vitro*, CASC2 overexpression dramatically repressed HCC cell proliferation and inhibited cell apoptosis; *in vivo*, CASC2 overexpression inhibited subcutaneous xenotransplant tumor growth. CASC2 affected the caspase cascades and NF-κB signaling in TRAIL-sensitive [Huh-7 (S) and HCCLM3 (S)] or TRAIL-resistant cell lines [Huh-7 (R) and HCCLM3 (R)] in different ways. In Huh-7 (S) and HCCLM3 (S) cells, CASC2 affected cell apoptosis through the miR-24/caspase-8 and miR-221/caspase-3 axes and the caspase cascades. miR-18a directly targeted CASC2 and RIPK1. In Huh-7 (R) and HCCLM3 (R) cells, CASC2 affected cell proliferation through the miR-18a/RIPK1 axis and the NF-κB signaling (**Figure 10**). RELA bound to CASC2 promoter region and inhibited CASC2 transcription.

The tumor suppressor effects of CASC2 on cancers were well-known previously. CASC2 binds to miR-362-5p and the downstream NF-κB signaling to inhibit the capacity of HCC cells to proliferate, migrate, or invade (44). CASC2 inactivates Wnt/beta-catenin signaling to directly downregulate miR-183, thereby inhibiting the viability and the colony formation, migratory, and invasive capacities of HCC cells (45). Through targeting miR-222, CASC2 attenuates HCC resistance to cisplatin (42). Herein, the abnormal downregulation of CASC2 in HCC tissues and cell lines was confirmed again. *In vitro*, CASC2 overexpression dramatically repressed HCC cell proliferation and inhibited cell apoptosis; *in vivo*, CASC2 overexpression inhibited subcutaneous xenotransplant tumor growth, confirming the tumor-suppressive role in HCC. More importantly, we demonstrated the miR-24/caspase-8 and miR-221/caspase-3 axes modulating HCC cell resistance to TRAIL treatment (26). Given these previous findings, it is necessary to investigate the underlying mechanisms of CASC2 affecting HCC sensitivity and resistance to TRAIL treatment.

As aforementioned, TRAIL could act on both apoptotic and non-apoptotic signaling. In TRAIL-sensitive cell lines, TRAIL activates caspase-8 by binding to receptor DR4/DR5, triggering the activation of caspase cascade signaling pathway and promoting apoptosis. Moreover, caspase-8 promotes the cleavage of RIPK1, inhibiting cell survival caused by NF-κB activation (12, 46, 47). In TRAIL-resistant cell lines, TRAIL recruits TRADD/TRAFs through the receptor DR4/DR5 to activate the NF-κB pathway and promote cell proliferation (48–50). Interestingly, in the present study, the increases in RIPK1, caspase-8, and caspase-3 in TRAIL-sensitive Huh-7 (S) and HCCLM3 (S) cells and increases in IKKβ, p-IκBα, and p-p65 in TRAIL-resistant Huh-7 (R) and HCCLM3 (R) cells were observed under TRAIL treatment, suggesting that TRAIL indeed acts on TRAIL-sensitive and TRAIL-resistant cells through different signaling pathways. As expected, in TRAIL-sensitive Huh-7 (S) and HCCLM3 (S) cells, CASC2 modulated caspase-8 and caspase-3 levels, whereas in TRAIL-resistant Huh-7 (R) and HCCLM3 (R) cells, CASC2 modulated IKKβ, p-IκBα, and p-p65 levels, indicating that CASC2 also affects TRAIL-sensitive and TRAIL-resistant HCC cells in different ways.

Increasing evidence support the hypothesis that lncRNA serves as ceRNA for miRNAs, counteracting miRNA-mediated repression on downstream mRNAs (51–53). More importantly, it has been revealed that numerous miRNAs were involved in HCC TRAIL resistance. For example, miR-26b targeted Mcl-1 in HCC cells to modulate TRAIL-induced cell apoptosis (21). miR-221 and miR-222 modulate the resistance to TRAIL and promote tumorigenesis *via* downregulating PTEN and TIMP3 in both aggressive non-small-cell lung cancer and hepatocarcinomatous cells (22). Given that the caspase cascades are activated in TRAIL-sensitive Huh-7 (S) and HCCLM3 (S) cells, and that CASC2 modulates caspase-8 and caspase-3 levels in TRAIL-sensitive Huh-7 (S) and HCCLM3 (S) cells, CASC2 might act on TRAIL-sensitive cells through the miR-24/caspase-8 and miR-221/caspase-3 axes reported in our previous study (26). As expected, in Huh-7 (S) and HCCLM3 (S) cells, CASC2 knockdown inhibited, whereas miR-24 inhibition or miR-221 inhibition promoted cell apoptosis and caspase-8/caspase-3 levels, the effects of CASC2 knockdown were significantly attenuated by miR-24 inhibition or miR-221 inhibition. Thus, CASC2 affects apoptotic signaling in TRAIL-sensitive cells through the apoptotic miR-24/caspase-8 and miR-221/caspase-3 axes and caspase cascades.

miRNAs interact with the 3’-UTR of target mRNAs to induce mRNA degradation or translational inhibition. Given the key role of RIPK1, a regulator of numerous programmed cell-death pathways and inflammation and a cell death mediator in hepatocarcinogenesis (54–58), online tools were used to analyze miRNAs that might target CASC2 and RIPK1, and miR-18a was selected. Altered miR-18a expression has been found in various physiological and pathological processes, including cell proliferation, apoptosis, epithelial-mesenchymal transition (EMT), tumorigenesis, cancer invasion, and metastasis (59). miR-18a has a dual functional role in different cancer types; in cancers of lung, gastric, cervical, and prostate, miR-18a serves as an oncogenic miRNA (59). In the present study, miR-18a targeted RIPK1 3’-UTR and inhibited RIPK1 expression. Similar to CASC2 knockdown, RIPK1 knockdown in TRAIL-resistant Huh-7 (R) and HCCLM3 (R) cells also significantly promoted HCC proliferation. As we have mentioned, in addition to the canonical apoptotic signaling pathway, TRAIL could also activate NF-κB, RIPK1, and TRAF2 signal pathways to be involved in non-canonical signaling pathway (60). Consistent with previous study, CASC2 or RIPK1 knockdown in TRAIL-resistant Huh-7 (R) and HCCLM3 (R) cells significantly inhibited NF-κB signaling activation; on the contrary, miR-18a knockdown activated the NF-κB signaling. Thus, in TRAIL-resistant Huh-7 (R) and HCCLM3 (R) cells, CASC2 modulates HCC proliferation through the miR-18a/RIPK1 axis and the NF-κB signaling. More importantly, according to the ChIP-Atlas database and experimental investigation, RELA targeted the promoter region of CASC2, inhibiting CASC2 transcription and forming a regulatory loop with the NF-κB signaling, modulating TRAIL-resistant Huh-7 (R) and HCCLM3 (R) cell proliferation through the non-apoptotic signaling.

In conclusion, CASC2 affects cell growth mainly *via* the miR-24/caspase-8 and miR-221/caspase-3 axes in TRAIL-sensitive HCC cells; while in TRAIL-resistant HCC cells, CASC2 affects cell growth mainly *via* miR-18a/RIPK1 axis and the NF-κB signaling. These occurrences forebode that regulating with lncRNA CASC2 expression level could be deemed as a newfangled strategy for the precaution and treatment of HCC and related pathological processes.

## Data Availability Statement

The original contributions presented in the study are included in the article/**Supplementary Material**. Further inquiries can be directed to the corresponding authors.

## Ethics Statement

The studies involving human participants were reviewed and approved by the Research Ethics Committee of the Third Xiangya Hospital. The patients/participants provided their written informed consent to participate in this study. The animal study was reviewed and approved by the ethics committee of the Third Xiangya Hospital.

## Author Contributions

JS, HX, XJ, and ZY designed the research and wrote the manuscript. ZLe performed the majority of experiments. ZLi collected and analyzed the clinical experimental data. HZ and ZD assisted with the animal experiments. XY performed the figures and the statistical analyses. All authors contributed to the article and approved the submitted version.

## Funding

This work was supported by the National Natural Science Foundation of China (Grant No. 81803085) and Natural Science Foundation of Hunan Province, China (Grant Nos. 2020JJ4919, 2019JJ40414, and 2018JJ2612).

## Conflict of Interest

The authors declare that the research was conducted in the absence of any commercial or financial relationships that could be construed as a potential conflict of interest.

## Publisher’s Note

All claims expressed in this article are solely those of the authors and do not necessarily represent those of their affiliated organizations, or those of the publisher, the editors and the reviewers. Any product that may be evaluated in this article, or claim that may be made by its manufacturer, is not guaranteed or endorsed by the publisher.
