# Supplementary material for: The lncRNA CASC2 Modulates Hepatocellular Carcinoma Cell Sensitivity and Resistance to TRAIL Through Apoptotic and Non-Apoptotic Signaling
Source: Front Oncol. 2022 Jan 25;11:726622. doi: 10.3389/fonc.2021.726622 (PMC8823509; doi:10.3389/fonc.2021.726622)
Supplement: Supplementary file 1 [file Table_1.docx]

**Table S1. the primer sequences for the study.**

| **Name** | **Forward (5’~3’)** | **Reverse (5’~3’)** |
| --- | --- | --- |
| RT-PCR  CASC2 | GCACATTGGACGGTGTTTCC | CCCAGTCCTTCACAGGTCAC |
| RT-PCR  RIPK1 | TGGGCGTCATCATAGAGGAAG | CGCCTTTTCCATGTAAGTAGCA |
| RT-PCR  Caspase-8 | AGAGTCTGTGCCCAAATCAAC | GCTGCTTCTCTCTTTGCTGAA |
| RT-PCR  Caspase-3 | GAAATTGTGGAATTGATGCGTGA | CTACAACGATCCCCTCTGAAAAA |
| RT-PCR  β-actin | TTCCAGCCTTCCTTCCTGGG | TTGCGCTCAGGAGGAGCAAT |
| RT-PCR  MiR-24-3p | RT: GTCGTATCCAGTGCGTGTCGTGGAGTCGGCAATTGCACTGGATACGACCTGTTC  F: GCTGGCTCAGTTCAGCAG | R: CAGTGCGTGTCGTGGA |
| RT-PCR  MiR-18a-5p | RT: GTCGTATCCAGTGCGTGTCGTGGAGTCGGCAATTGCACTGGATACGACCTATCT  F: GCCTAAGGTGCATCTAGTGC | R: CAGTGCGTGTCGTGGA |
| RT-PCR  MiR-221-3p | RT: GTCGTATCCAGTGCGTGTCGTGGAGTCGGCAATTGCACTGGATACGACGAAACC  F: GCAGCTACATTGTCTGCTG | R: CAGTGCGTGTCGTGGA |
| RT-PCR  U6 | CTCGCTTCGGCAGCACA | AACGCTTCACGAATTTGCGT |
| RT-PCR  CASC2 promoter | TAACTGAACAAAACACCAACACTAT | ATCTTCAGTGGTCTTTTCAGTATG |
| Sh-CASC2 | GATCCGGCAGATGGAGATTCAGAAACACTCGAGTGTTTCTGAATCTCCATCTGCTTTTTG | AATTCAAAAAGCAGATGGAGATTCAGAAACACTCGAGTGTTTCTGAATCTCCATCTGCG |
| Sh-RIPK1 | GATCCGGCCAACCTCAAGTACTGTATC CTCGAGGATACAGTACTTGAGGTTGGC TTTTTG | AATTCAAAAAGCCAACCTCAAGTACTGTATCCTCGAGGATACAGTACTTGAGGTTGGCG |
| Sh-RELA | GATCCGGAGCACAGATACCACCAAGACTCGAGTCTTGGTGGTATCTGTGCTCCTTTTTG | AATTCAAAAAGGAGCACAGATACCACCAAGACTCGAGTCTTGGTGGTATCTGTGCTCCG |
| Sh-NC | GATCCACACAGCAGGTCAAGAGGAGTCTCGAGACTCCTCTTGACCTGCTGTGTTTTTTG | AATTCAAAAAACACAGCAGGTCAAGAGGAGTCTCGAGACTCCTCTTGACCTGCTGTGTG |
| PcDNA.3.1-CASC2 overexpression Vector construction primer | ctagcgtttaaacttaagcttAGCGGGCTGCAGGGCTGC | tgctggatatctgcagaattcTTTTTTTTTTTTGTTTTGCATGAAA |
| PcDNA.3.1-RIPK1 overexpression Vector construction primer | ctagcgtttaaacttaagcttATGTGGAGCAAACTGAATAATGAAGA | tgctggatatctgcagaattcTTAGTTCTGGCTGACGTAAATCAAG |
| Wt-CACS2  Vector construction primer | aattctaggcgatcgctcgagAGGTTAAACAGTGAGAAACAGCAAAG | attttattgcggccagcggccgcTATTTATAGGGTTGTTCACCAAAGGG |
| mut-CACS2  Vector construction primer | CAATAAtacgtggaatTATTTGTATAGCTATACACACTAATTTACATGATC | TAattccacgtaTTATTGTTATTCAATTTTATATTAGATTATGGC |
| Wt-RIPK1 3’UTR  Vector construction primer | aattctaggcgatcgctcgagCCCCACCTACTAGATGCCAGG | attttattgcggccagcggccgcTTTGATAACACCATTTGGATGGTG |
| mut-RIPK1 3’UTR  Vector construction primer | TCTCgatagacgtgCTGTCCGGTTACTACTTGGCCA | ACAGcacgtctatcGAGAGCCGAGGCTCATTACTTTA |
| Wt-CACS2 promoter  Vector construction primer | aattctaggcgatcgctcgagAAGAGAGAGTCGTTTTAAAATGTAAAGAA | attttattgcggccagcggccgcTTAATTTAAAAAATCATAATATAAATTAACACTAGG |
| mut-CACS2 promoter  Vector construction primer | GGAAATaagggacttcCTTAGAAGTTCAACCAGTATTTTTCTCTC | AGgaagtcccttATTTCCAAAATAGTGTTGGTGTTTTG |
| MiR-18a-5p mimics | UAAGGUGCAUCUAGUGCAGAUAG | AUCUGCACUAGAUGCACCUUAUU |
| MiR-18a-5p inhibitor | CUAUCUGCACUAGAUGCACCUUA |  |
| MiR-24-3p mimics | UGGCUCAGUUCAGCAGGAACAG | GUUCCUGCUGAACUGAGCCAUU |
| MiR-24-3p inhibitor | CUGUUCCUGCUGAACUGAGCCA |  |
| MiR-221-3p mimics | AGCUACAUUGUCUGCUGGGUUUC | AACCCAGCAGACAAUGUAGCUUU |
| MiR-221-3p inhibitor | GAAACCCAGCAGACAAUGUAGCU |  |
| Mimics NC | UUCUCCGAACGUGUCACGUTT | ACGUGACACGUUCGGAGAATT |
| Inhibitor NC | CAGUACUUUUGUGUAGUACAA |  |
