# Supplementary material for: The lncRNA CASC2 Modulates Hepatocellular Carcinoma Cell Sensitivity and Resistance to TRAIL Through Apoptotic and Non-Apoptotic Signaling
Source: Front Oncol. 2022 Jan 25;11:726622. doi: 10.3389/fonc.2021.726622 (PMC8823509; doi:10.3389/fonc.2021.726622)
Supplement: Supplementary file 2 [file Table_2.docx]

**Table S2. The transcription factor binding sites of lncRNA CASC2 gene promoter region in liver cancer tissues based on ChIP-ATLAS (https://chip-atlas.org/)**

| ID | Antigen | Cell | Num of peaks | Overlaps/ Control | Log P-val | Fold Enrichment |
| --- | --- | --- | --- | --- | --- | --- |
| [SRX2270127](http://chip-atlas.org/view?id=SRX2270127) | RELA | HuH-7 | 734 | 149/18550 | 0.0 | 99999 |
| [SRX2270128](http://chip-atlas.org/view?id=SRX2270128) | RELA | HuH-7 | 589 | 267/18550 | 0.0 | 99999 |
| [SRX2270129](http://chip-atlas.org/view?id=SRX2270129) | RELA | HuH-7 | 2572 | 1367/18550 | 0.0 | 99999 |
