# Supplementary material for: The lncRNA CASC2 Modulates Hepatocellular Carcinoma Cell Sensitivity and Resistance to TRAIL Through Apoptotic and Non-Apoptotic Signaling
Source: Front Oncol. 2022 Jan 25;11:726622. doi: 10.3389/fonc.2021.726622 (PMC8823509; doi:10.3389/fonc.2021.726622)
Supplement: Supplementary file 3 [file Table_3.docx]

**Table S3. The** **predicted one RELA binding sites with high score (score > 8) in lncRNA CASC2 promoter regions based on JASPAR (http://jaspar.genereg.net/)**

| **Matrix ID** | **Name** | **Score** | **Relative Score** | **Start** | **End** | **Predicted sequence** |
| --- | --- | --- | --- | --- | --- | --- |
| MA0107.1 | RELA | 10.3164 | 0.870390291984 | 578 | 587 | AGGACTTTCC |
| MA0107.1 | RELA | 9.77443 | 0.856206896749 | 1655 | 1664 | TGGACTTCCC |
| MA0107.1 | RELA | 8.14291 | 0.813512739605 | 879 | 888 | TTGATTTTCC |
